# The miRNA 196a2 rs11614913 variant has prognostic impact on Turkish patients with multiple myeloma

**DOI:** 10.1186/s13104-020-05392-9

**Published:** 2020-11-23

**Authors:** Melya Pelin Kirik, Mustafa Pehlivan, Ayse Feyda Nursal, Yasemin Oyaci, Sacide Pehlivan, Istemi SERIN

**Affiliations:** 1 Department of Hematology, Faculty of Medicine, Gaziantep University, Gaziantep, Turkey; 2 Department of Medical Genetics, Faculty of Medicine, Hitit University, Corum, Turkey; 3 Department of Medical Biology, Faculty of Medicine, Istanbul University, Istanbul, Turkey; 4 Department of Hematology, Istanbul Training and Research Hospital, University of Health Sciences, Org.Nafiz GURMAN CAD. 34098, Istanbul, Turkey

**Keywords:** Multiple myeloma, *miR*-*196a2*, Autologous stem cell transplantation, Prognosis

## Abstract

**Objective:**

Multiple myeloma (MM) arises from malignant plasma cells as a single clone in the bone marrow. Accumulating evidences have reported that there is an association between *miR*-*196a2* (rs11614913) variant and various cancers while there were unverified and inconsistent results in MM. The goal of this study is to investigate the impact of the *miR*-*196a2* variant on clinical findings and susceptibility in MM. Two hundred MM patients (156 patients under transplantation of autologous stem cell) and 200 healthy controls included in this study.

**Results:**

The statistical analysis showed no significant relationship for allele and frequencies of *miR*-*196a2* genotype between patients and controls (*p *> *0.05*). Log-rank test showed that gender has highly significant impact on both OS and PFS (*p *= *0.027, p *= *0.045).* In the univariate analysis, TT genotype (*p *= *0.022*), and CT/TT (*p *= *0.008*) had better OS. In the multivariate analysis, CC/CT-TT were associated with positively OS (*p *= *0.041*). Currently, the most valuable prognostic markers in MM that has clinical implication are genetic abnormalities. It can be concluded from the results that *miR*-*1962a* variant is effective in prognosis of the MM. It is believed that these findings will help us understand the molecular basis of disease.

## Introduction

Multiple myeloma (MM) arises out of malignant plasma cells (PCs) as a single clone in the bone marrow and is the second most common hematological cancer [1]. MM has the incidence of 0.5–1/100,000 in Asia, while the incidence in America and Africa varies between 10 and 12/100,000 [2]. Usually, the MM patients had the median age of 69, and majority them (two-thirds) are male [3]. The median survival time of MM patients has improved in the last 20 years from 3 to 6 years due to developments in treatments. Currently, the MM therapy involves the combined administration of multiple chemotherapeutic agents and autologous stem cell transplantation (ASCT) [4].

MicroRNAs (miRNAs), 25-nucleotide long noncoding RNAs, have been assumed to be crucial since the expression of target genes can be negatively regulated at the posttranscriptional level through binding to their 3′-untranslated regions (3′-UTRs) [5, 6]. The main tasks of miRNAs are apoptosis, proliferation, immune response, differentiation and inflammation. The miRNA expression and/or maturation can be modified for the SNPs presenting in the miRNA gene region to influence the features of miRNA, resulting in abnormal miRNA regulation [7]. There is microRNA-196a (*miR*-*196a*) between HOXC9 and HOXC10 on chromosome 12 (12q13.13) [8]. The *miR*-*196a2* rs11614913, as a definitional miRNA variant, extends in the miR-196a2 mature sequence, negatively affecting miRNA precursor endogenous processing maturely. According to Hoffman et al., the transcription level of mature miR-196a was not only influenced by the *miR*-*196a2* rs11614913, but also target gene generation was biologically affected [9]. There is a significant relationship between *miR*-*196a2* rs11614913 and cancer risk [10].

It has been shown that miRNAs act as tumor suppressors by suppressing oncogene expression or they can act as oncogenes by suppressing other tumor suppressors. A global reduction in miRNA processing promotes carcinogenesis and miRNA profiling data has been successfully applied to classify tumors and predict prognosis in a number of cancer types [7, 9]. Although there is no literature data in MM, by looking at studies in the solid tumors in which the relationship between miRNA and malignancy is discussed, it can be said that it has an opportunity to play a role in the development of the disease through the mechanism of altering the prooncogene-tumor suppressor gene balance in MM.

To best of our knowledge there are no studies on the effect of *miR196a2* variants on risk of MM on till date. Therefore, the goal is to investigate the impact of the *miR*-*196a2* rs11614913 variant on clinical characteristics and susceptibility in MM patients.

## Main text

### Materials and methods

#### Study population

Total of 200 patients (109 males, 91 females, mean age: 56 years old) who were diagnosed and treated with MM, at the Division of Hematology, Gaziantep University Faculty of Medicine, (Gaziantep, Turkey) between April 2018–January 2019 included in this case–control study. One hundred and fifty-six patients underwent ASCT and received bortezomib, cyclophosphamide, dexamethasone (VCd) by induction. Melphalan was given as preparation regimen for ASCT. Rd (Lenolidomide, dexamethasone) treatment was given as maintenance treatment. The demographical findings such as age, gender, and clinical data including diagnosis stage (DS, ISS), ECOG performance score, treatment agents, treatment response, transplantation data, survival data, duration of follow and laboratory findings at diagnosis [C-reactive protein (CRP),complete blood count (CBC), β2 microglobulin, total protein, albumin, globulin, lactate dehydrogenase (LDH), creatinine, cytogenetic results, immunoglobulin subtypes, FISH analysis] were evaluated retrospectively by medical file review. It was decided whether there was bone involvement based on direct X-ray, MRI, PET-CT or CT scan. A total of 200 healthy controls (99 females, 101 males, mean age of 53 years) were recruited from the Internal Medicine Clinics in physical examination. Controls were matched with patients by sex and age. It was confirmed that all control subjects had no malignancy. Ethical committee approval was received (Gaziantep University-2018/78) and the patients and control subjects gave the informed consent before beginning of the study. The experimental procedures were based on the Declaration of Helsinki and relevant institutional regulations.

#### Genotyping

The intravenous blood (5 ml) was collected from subjects in EDTA vacutainers. the commercial kit (Genemark, Plus Blood Genomic Purification Kit, USA) was used to extract the genomic DNA based on the manufacturer instructions. The polymerase chain reaction-restriction fragment length polymorphism (PCR–RFLP) was used for the genotype analysis of the *miR*-*196a2* rs11614913 variant as described by Bodal et al. [11].

#### Statistical analysis

The Statistical Package for the Social Sciences (SPSS) for Windows (version 20.0; SPSS Inc, Chicago, IL, USA) was used to analyze all data. Mean ± SD and minimum/maximum were given to show continuous data. Significance of differences in the allele frequency and genotype distribution between the groups was measured using the χ2 test. 95% confidence intervals (CI) and odds ratio (OR) were calculated. *p *≤* 0.05* was considered statistically significant.

## Results

Total of 200 MM patients and 200 controls were included in this study. In the MM patients group, 91 patients (45.5%) were females and 109 (54.5%) were males. There were 99 (49.5%) females and 101 (50.5%) males in the control group. Table 1 shows the patients and controls’ clinical and demographical characteristics.

**Table 1:** Demographic and clinical characteristics of groups

		Patient group		Control group
		Median	N:200 (%)	N:200 (%)
Age			56 (28-82)	53 (22-68)
Gender	Female/Male		91/109 (45.5/54.5)	99/101 (49.5/50.5)
Ig subtypes	κ/λ		110/60 (64.7/35.3)	
	G/A		112/30 (65.2/17.4)	
	Light Chain		30 (17.4)	
Stages (Salmon-Durie)	II/III		53/117 (31/69)	
	A/B		129/41 (76/24)	
ISS	I		52 (30.4)	
	II/III		44/75 (25.7/43.9)	
ECOG	>1		32/179 (17.9)	
Hemoglobin	gr/dl	10.3 (5.9-15.5)		
White blood cell count	/μl	6985 (5900-17,000)		
Platelet count	10^3^/μl	170 (28-788)		
C-reactive protein	mg/dl	8.8 (2.1-352)		
LDH	IU/l	203 (93-1037)		
β2-microglobulin	mg/l	5.0 (1.5-47)		
Albumin	g/l	3.5 (1.6-5.1)		
Treatment	VCd, OHKHT, Rd		156 (78)	
	VCd ± Rd		44 (22)	
Cytogenetic results (n:190)	Normal (XX, XY)		179	
	13q		2	
	Monosomy (X, 14-22)		2	
	Hyper/diploidy		2 + 2	
	t (4;11)		1	
	t (4;14)		1	
	Trisomy 7		1	
OS (5 years) %		71		
PFS (months)^a^		43.8		
Mortality			47 (26.5)	
Follow-up period ^a^(month)		30 (4.8-155)		

*VCd* Bortezomib, Cyclophosphamide, dexamethasone, *Rd* Lenolidomide, dexamethasone, *AHSCT* autologous hematopoeticstem cell transplantation, *ECOG* performance scores, *Ig* Immunglobulin, *LDH* Lactate dehydrogenase, *ISS* internationale stage system, *OS* overall survival, *PFS* Progression free survival

^a^median

ASCT treatment was administered to 156 patients. Table 2 gives the clinical findings of these patients undergoing ASCT.

**Table 2:** Clinical findings of patients underwent ASCT

		Patients		Controls
			N:156 (%)	N:200 (%)
Age			55 (28–72)	53 (22–68)
Gender	Female/Male		75/81 (48/52)	99/101 (50/50)
Diagnosis age^a^ (years)			55 (28–72)	
Ig subtypes	κ/λ		88/40 (68/32)	
	G/A		84/20 (66/16)	
	Light chain		24 (18)	
Stage (Salmon-Durie)	II/III		53/89 (38/62)	
	A/B		97/31 (76/24)	
ISS	I		42 (33)	
	II/III		35/52 (28/41)	
ECOG	>1		12/127 (9.4)	
Hemoglobin	gr/dL	10.4 (6.2–15)		
White blood cell count	/μL	6980 (2700–18,500)		
Platelet count	10^3^/μl	162 (61–406)		
C-reactive protein	mg/dl	7.4 (2.1–352)		
LDH	IU/l	208 (93-1037)		
β2-microglobulin	mg/l	4.9 (1.5–47)		
Albumin	g/l	3.5 (1.6–5.1)		
Treatment	VCd, AHSCT, Rd		156 (100)	
OS (5 years) %		79		
PFS^a^		55.3		
Mortality			25 (16)	
Follow-up period ^a^(month)		36 (7.8-155)		

*AHSCT* Autologous hematopoietic stem cell transplantation, *VCd* Bortezomib, Cyclophosphamide, dexamethasone, *Rd* Lenolidomide, dexamethasone, *ECOG* Eastern Cooperative Oncology Group, *Ig* Immunglobulin, *LDH Lactate dehydrogenase*, *ISS* internationale stage system, *OS* overall survival, *PFS* Progression free survival

^a^median

Table 3 gives the genotype and allele distributions of the MM patients and healthy controls for the *miR*-196a2 rs11614913 variant. The groups did not show any statistically significant difference in terms of *miR*-*196a2* rs11614913 allele and genotype frequencies (p > 0.05).

**Table 3:** The allele and genotype distributions of *miR*-*196a2* rs11614913 in groups

*MiR*-*196a2* rs11614913	Patient group	Control group	OR Exp(B)	95% CI	P
Genotypes	n:200 (%)	n:200 (%)			
CC	79 (39.5)	68 (34)	0.976^a^	0.525–1.815^a^	0.939^a^
CT	91 (45.5)	106 (53)	1.317^a^	0.724–2.395^a^	0.367^a^
TT	30 (15)	26 (13)	1.080^&^	0.617–1.891^b^	0.887^b^
Alleles					
C	249 (62.25)	242 (60.5)	0.933^b^	0.702–1.240^b^	0.663^b^
T	151 (37.75)	158 (39.5)			

^a^OR (95%CI) corrected according to gender and age

^b^Fisher’s Exact Test

The relationships between *miR*-*196a2* rs11614913 and MM patients’ survival were analyzed in different genetic models such as dominant and codominant models using the Cox regression analyses **(**Additional file 1: Table S1). Three factors that determines better PFS in univariate analysis were female gender (*p *= *0.045*), ECOG (≤ 1) (*p *= *0.029*), and Vcd, Rd (*p *= *0.001*).

The nine factors that determine better OS were females (*p *= *0.027*), younger age (< 65) (*p *= *0.001*), ISS III versus ISS I and II (*p *= *0.009*), ISS III versus ISS I/ISS II (*p *= *0.001*), Ig λ (*p *= *0.025*), VCd, Rd (*p *= *0.001*), *miR*-*196a2* TT genotype (*p *= *0.022*) and CT/TT genotypes (*p *= *0.008*) from the univariate analysis.

Cox proportional hazards regression was used for multivariate analysis to identify prognostic markers for survival (Additional file 1: Table S2). There was relationship between ECOG > 1 (*p *= *0.016*), ISSI/II-III (*p *= *0.045*) and miR196a2 CC/CT-TT (*p *= *0.041*) and OS. Additionally, ASCT as first line treatment was positively related to PFS (*p *= *0.001*).

## Discussion

MM arising out of the post-germinal lymphoid B cell lineage is a neoplasm of clonal plasma cells, which occurs after commitment to a lineage in the bone marrow of progenitor cells [12]. Comprehending the prognostic factors in MM is crucial for optimal management of MM patients. In this study, the goal was to ascertain whether *miR*-*196a2* rs11614913 variant affects susceptibility and prognosis in MM. The results indicate that *miR*-*196a2* rs11614913 have a prognostic impact in MM in a Turkish population.

MiRNAs hindering the expression of protein coding genes using translational repression or mRNA degradation are the non-coding. Different genes under different conditions in a specific cell type or in various cell types may be modulated by the *miR*-*196a2*. Based on the analysis of the genotype–phenotype correlation, CC homozygote in *miR*-*196a2* was linked with expression of *miR*-*196a2*, which was enhanced significantly [13]. There is rs11614913 in the 3′ passenger strand mature sequence of *miR*-*196a*. The allele frequencies which are indicator of genetic diversity in a population indicate the gene variants frequency. The meta-analysis reported that the *miR*-*196a* rs11614913 variant allele frequencies showed differences between distinct ethnic groups. There are inconsistent results obtained from analysis of subgroup based on ethnicity which may be shown partially with the difference in allele frequencies among races [14]. This study found C allele as the major allele in both patient and control group.

The relationship between susceptibility to cancer and *miR*-*196a2* rs11614913 variant has been investigated by several investigators. Li et al. reported an association between a higher risk of gastric cancer in a Chinese population and the CC genotype of rs11614913 [15]. Tian et al. reported an association between the homozygote CC of *miR*-*196a2* rs11614913 variant and risk of lung cancer increase by about 25% as compared to their heterozygote TC and wild-type homozygote TT [16]. The meta-analysis showed *miR*-*196a2* rs11614913 variant to be associated with susceptibility to cancer, particularly head and neck cancer, hepatocellular carcinoma, and lung cancer [17]. In a study investigating 15 case–control study, there was a higher risk of cancer in those who had the genotypes of TC/CC than that in those who had genotype of TT [18]. Wang et al. showed increase of the cancer risk in homozygote comparison model due to *miR*-*196a2* rs11614913 CC genotype as compared to TT genotype [19]. In a meta-analysis, authors concluded that there was significant linkage between the *miR*-*196a2* rs11614913 and a reduced risk of cancer, especially a lower risk of gastric cancer and colorectal cancer or for Asian subgroup of population [20]. Furthermore, in the meta-analysis, Xu et al. reported the most probable contribution of the rs11614913 reduces cancer susceptibility, particularly breast cancer in Asia [21]. *miR*-*196a2* rs11614913 T allele was shown to have significant relationship with reduced risk of breast cancer [22]. Also, the *miR*-*196a2* rs11614913 TT genotype was demonstrated to have significant relationship with a lower risk of cancer particularly lower risk of lung and colorectal cancer among the population subgroup in Asia [23].

There are only few studies have been conducted on the relationship between *miR196*-*a2* rs11614913 variant and the hematological malignancies. There has been description of abnormal function of expression of miRNA expression for almost all lymphomas and the possibility for miRNAs to be used as diagnostic biomarkers is emphasized by the distinct miRNA signatures [24]. The target genes of *miR*-*196a2* were revealed to constitute Hox genes which are a homeobox family of genes. They are effective in formation of differentiation and growth of hematopoietic cell. Some evidences showed HOXC8 suppressing metastasis and migration of cell to be the target gene of *miR*-*196a2*  [25]. Rakmanee et al. reported significant relationship between the CC/TC genotypes and *miR196*-*a2* rs11614913 CC, TC heterozygote and susceptibility to the increased acute lymphoblastic leukemia (ALL) during childhood as compared to the TT wild type [26]. In a study by Li et al., the relationship between the non-Hodgkin lymphoma risk and *miR*-*196a2* rs11614913 was evaluated among 320 healthy controls and 318 NHL cases in a case–control study [27]. They found a relationship between the combined TC/CC genotypes and bone marrow invasion, B symptoms and Ann Arbor stage. Deghady et al. revealed that the CC genotype and C allele were significantly prevalent in patients with chronic lymphocytic leukemia (CLL) [8]. They showed that the C allele carriers had 3.38 fold increase in risk of CLL development regarding the protection of T allele against CLL.

This study is the first one which investigates *miR*-*1962a* rs3217927 in Turkish patients suffering from MM. There was found no relationship in allele and genotype distribution of *miR*-*196a2* rs11614913 between patients and controls in our samples. Comparing TT genotype to CC and CT genotypes, the subjects carrying TT genotype had significantly higher OS (*p˂0.05*). *Also, miR*-*196a2* CT/TT genotypes were significantly correlated with higher OS.

## Conclusion

Comprehension of the prognostic factors in MM is crucial for the ideal management of MM patients. Currently, the most valuable prognostic markers in MM that has clinical implication are genetic abnormalities. It can be concluded from the results that *miR*-*1962a* rs3217927 variant is effective in prognosis of the MM in Turkish patients. We believe these findings will help us to understand the molecular basis of disease. However, further studies should be conducted on different ethnicities and larger population.

## Limitations

Our study had certain limitations. It is necessary to say that the limited number of patients is effective especially in genotype subgroups where no significant statistical difference can be detected.

## Supplementary information

**Additional file 1: Table S1.** The analysis of prognostic factors in all patients with MM (Univariate analysis, Logrank test). **Table S2.** Prognostic factors of MM patients (Cox proportional hazard model backward ,Multivariate analysis).

## Data Availability

The datasets analysed during the current study are available on [figshare], under 10.6084/m9.figshare.13229141.v1.

## Abbreviations

miRNA: Micro ribonucleic acid
OS: Overall survival
PFS: Progression free survival
MM: Multiple myeloma
PCR-RFLP: Polymerase Chain Reaction–restriction fragment length polymorphisms
ASCT: Autologous stem cell transplantation
3′-UTRs: 3′-Untranslated regions
VCd: Bortezomib, cyclophosphamide, dexamethasone
Rd: Lenalidomide, dexamethasone
CRP: C- reactive protein
CBC: Complete blood count
LDH: Lactate dehydrogenase
FISH: Fluorescence in situ hybridization
MRI: Magnetic resonance imaging
PET-CT: Positron emission tomography-computed tomography
CI: Confidence interval
OD: Odd RATIO
ISS: International staging system
ALL: Acute lymphoblastic leukemia
CLL: Chronic lymphoblastic leukemia
ECOG: Eastern cooperative oncology group
