# Supplementary material for: The miRNA 196a2 rs11614913 variant has prognostic impact on Turkish patients with multiple myeloma
Source: BMC Res Notes. 2020 Nov 23;13:545. doi: 10.1186/s13104-020-05392-9 (PMC7682760; doi:10.1186/s13104-020-05392-9)
Supplement: Supplementary file 1 — Additional file 1: Table S1. The analysis of prognostic factors in all patients with MM (Univariate analysis, Logrank test). Table S2. Prognostic factors of MM patients (Cox proportional hazard model backward ,Multivariate analysis). [file 13104_2020_5392_MOESM1_ESM.docx]

**Table S1.** The analysis of prognostic factors in all patients with MM (Univariate analysis, Logrank test)

|  |  | **n** | **PFS**  **(5-years %)** | **Log Rank**  **p** | **OS**  **(5-years %)** | **Log Rank**  **p** |
| --- | --- | --- | --- | --- | --- | --- |
|  |  | 200 | 43.8 |  | 71 |  |
| **Gender** | Females/Males | 91/109 | 64.3 / 28.3 | **0.045** | 81 / 77.5 | **0.027** |
| **Age** | <65 / ≥65 | 149/51 | 52.7 / 21.8 | 0.133 | 88 / 42.3 | **0.001** |
| **Stage** | II/III | 53/117 | 43.8/24.7 | 0.514 | 87.1 / 99.1 | 0.205 |
|  | A/B | 129/41 | 72.2/28.5 | 0.101 | 99.1 / 82.9 | 0.061 |
| **ISS** | I | 52 | 64.3 |  | 87 |  |
|  | II | 44 | 39.2 |  | 68 |  |
|  | III | 75 | 28.5 | 0.393 | 56 | **0.009** |
| **ISS** | I/II | 96 | 47.1 |  | 78 |  |
|  | III | 75 | 28.5 | 0.176 | 56 | **0.003** |
| **Ig subtypes** | κ/λ | 110/60 | 52.7/33.9 | 0.164 | 76 / 87.1 | **0.025** |
|  | G | 113 | 39.2 |  | 72 |  |
|  | A | 28 | 43.8 |  | 54 |  |
|  | Light Chain | 29 | 52.7 | 0.412 | 73 | 0.261 |
| **ECOG** | ≤ 1 / >1 | 137/31 | 44.4 / 17.1 | **0.029** | 74 / 29.4 | **0.001** |
| **Platelet(x100/l)** | <150 / ≥150 | 18/84 | 23.5 / 24.4 | 0.798 | 59 /77.5 | 0.324 |
| **LDH (IU/l)** | <480 / ≥480 | 157/14 | 40.4 / 21.8 | 0.769 | 69 / 99.1 | 0.329 |
| **CRP (mg/l)** | <5 / ≥ 5 | 69/101 | 44.4 / 38.5 | 0.398 | 78 / 87.1 | 0.087 |
| **First-line tratment** | VCd, AHSCT, Rd | 156 | 54.3 |  | 79 |  |
|  | VCd, Rd | 44 | 11.4 | **0.001** | 29.4 | **0.001** |
| ***MiR-196a2*** | CC | 79 | 31.5 | 0.274 | 65 | **0.022** |
|  | CT | 91 | 67.1 |  | 72 |  |
|  | TT | 30 | 27.4 |  | 82 |  |
| ***MiR-196a2*** | CC | 79 | 31.5 | 0.098 | 65 | **0.008** |
|  | CT/TT | 121 | 67.1 |  | 75 |  |
| *Median (month), VCd: Bortezomib, Cyclophosphamide, dexamethasone; Rd: Lenolidomide, dexamethasone; AHSCT: autologous hematopoeticstem cell transplantation; ECOG: performance scores; Ig: Immunglobulin; LDH: *Lactate dehydrogenase*; ISS: internationale stage system; Stage: Durie Salmon; OS: overall survival*median; PFS: Progression free survival . The statistically significant results are shown in boldface. | | | | | | |

**Table S2.** Prognostic factors of MM patients (Cox proportional hazard model backward ,Multivariate analysis).

|  |  | **OS** |  |  | **PFS** |  |
| --- | --- | --- | --- | --- | --- | --- |
|  | **Exp (B)**  **Relative risk** | **%95 CI** | **p** | **Exp (B)**  **Relative risk** | **%95 CI** | **P** |
| **ECOG≤ 1 / >1** | 0.385 | 0.178-0.835 | **0.016** | 0.810 | 0.440-1.492 | 0.500 |
| ***MiR*-196a2**  **CC / CT-TT** | 0.541 | 0.281-0.973 | **0.041** | 0.825 | 0.527-1.292 | 0.401 |
| **First Line Tratment**  **ASCT +/-** | 0.541 | 0.251-1.167 | 0.117 | 0.414 | 0.250-0.687 | **0.001** |
| **Gender (Females/Males)** | 0.682 | 0.351-1.325 | 0.259 | 0.664 | 0.420-1.049 | 0.079 |
| **ISS I/II-III** | 0.515 | 0.265-1.003 | **0.045** |  |  |  |
| **DS Stage A/B** | 1.707 | 0.779-3.736 | 0.182 |  |  |  |
| **Age<65 / ≥65** | 0.714 | 0.351-1.452 | 0.353 |  |  |  |
| **CRP <5 / ≥ 5** | 0.643 | 0.320-1.293 | 0.216 |  |  |  |
| OS: overall survival; ECOG: performance scores; ISS: International staging system; PFS: progression-free survival; AHSCT: autologous hematopoetic stem cell transplantation; CRP: C-reactive protein; DS: Durie-Salmon staging; The statistically significant results are shown in boldface. | | | | | | |
